# Developments and Emerging Trends in the Global Treatment of Chronic Rhinosinusitis From 2001 to 2020: A Systematic Bibliometric Analysis

**DOI:** 10.3389/fsurg.2022.851923

**Published:** 2022-04-07

**Authors:** Fangwei Zhou, Tian Zhang, Ying Jin, Yifei Ma, Zhipeng Xian, Mengting Zeng, Guodong Yu

**Affiliations:** ^1^Department of Clinical Medicine, Guizhou Medical University, Guiyang, China; ^2^Department of Otorhinolaryngology Head and Neck Surgery, Affiliated Hospital of Guizhou Medical University, Guiyang, China

**Keywords:** chronic rhinosinusitis, treatment, bibliometric analysis, trend, CiteSpace, VOSviewer

## Abstract

**Background:**

Research on the treatment of chronic rhinosinusitis (CRS) has increased in recent decades. We undertook a bibliometric and visualization analysis of studies on CRS treatment to track research trends and highlight current research “hotspots”.

**Methods:**

Original publications related to CRS treatment were obtained from the Science Citation Index-Expanded (SCI-E) and Social Sciences Citation Index (SSCI) databases in the Web of Science Core Collection (WoSCC) of Clarivate Analytics between 2001 and 2020. The country/region, institution, author, journal, references, and keywords involved in this topic were extracted using CiteSpace and VOSviewer to identify and analyze the research focus and trends in this field.

**Results:**

In the previous two decades (especially after 2015), the number of publications on CRS treatment has grown markedly. With regard to publications and access to collaborative networks, the leading country was the USA. High-frequency keywords were “CRS,” “endoscopic sinus surgery,” “sinusitis,” “nasal polyps,” “asthma,” “rhinosinusitis,” “management,” “diagnosis,” “outcomes,” and “quality of life.” Inspection of keyword bursts suggested that “clinical practice guideline,” “adult CRS,” “innate lymphoid cell,” “recurrence,” and “mepolizumab” are the emerging research hotspots. The timeline view of the cluster map revealed that biologic agents have become an up-and-coming “hot topic” in CRS treatment in recent years.

**Conclusion:**

Academic understanding of CRS treatment has improved markedly over the past 20 years. We study analyzed the papers objectively, methodically, and comprehensively, and identified hotspots and prospective trends in the field of CRS treatment. These results will aid rhinologists in gaining greater insight into CRS treatment strategies and identifying the changing dynamics of CRS research.

## Introduction

Chronic rhinosinusitis (CRS) is a chronic inflammatory disease of the nose and paranasal sinuses lasting >12 weeks (1, 2). It is characterized primarily by chronic nasal obstruction, decreased olfaction, and facial discomfort (1). Large-scale epidemiological research has shown that CRS prevalence varies worldwide, reaching ≤8% in China, but >10% in the USA and Europe (2–4). Despite being readily treatable, CRS imposes social and economic burdens worldwide. CRS costs the USA billions of dollars in direct and indirect medical expenditures each year (5). In Europe, the direct costs for a CRS patient has been estimated to be 1,500 per year (6). The indirect costs of CRS are substantially lower than direct costs, such as missed workdays and reduced productivity at work, which increase the economic burden of the disease significantly (7).

Guidelines recommend diagnosing CRS based on sinus symptoms lasting ≥12 weeks and objective evidence of inflammation of the sinuses upon nasal endoscopy or computed tomography of paranasal sinuses because a diagnosis based on symptoms alone might be inaccurate (8). Academic understanding of CRS pathophysiology continues to evolve. Traditionally, CRS has been classified into two categories: CRS with nasal polyps (CRSwNP) and CRS without nasal polyps (CRSsNP). This classification obscures the underlying complex pathophysiologic mechanisms of CRS (9). CRS is a series of inflammatory conditions with varying degrees of overlap between non-type-2 inflammation and type-2 inflammation with eosinophilia (10).

Typically, the treatment strategy for CRS is combined primarily with reduction of mucosal inflammation, prevention of infection, and removal of mucus from the sinuses (11). Management options for CRS include endoscopic sinus surgery (ESS), antibiotics, nasal glucocorticoids, systemic corticosteroids, irrigations, and biologics (1, 12). Despite being treated with drugs and/or surgery, individuals with CRS frequently complain of symptom recurrence (13).

In recent years, numerous investigations on the immunological processes of CRS have revealed various novel endotypes that hold promise as biomarkers for targeted therapy of resistant CRS (14). The introduction of “precision medicine” to manage CRS is a step forward in delivering “tailored” therapy for all patients with CRS. During recent decades, scholars have made tremendous progress in understanding the epidemiology, diagnosis, pathophysiology, and treatment of CRS. Nevertheless, there is a scarcity of reports that synthesize different pieces of information that can aid researchers to acquire a comprehensive visual overview of research trends in CRS treatment.

Bibliometric analysis is a pioneering method used to evaluate quantitatively the impact of research literature on a selected research area over a given period, countries/regions, research collaboration, journals, institutions, and authors (15). Bibliometric analysis has emerged in various research domains (16). In contrast to traditional systematic reviews and meta-analyses, bibliometric analysis can reveal the current status and development of research topics more systematically and visually (17). Bibliometric analysis can also be used to identify current highlights and “hotspots” for researchers to generate ideas and perspectives to guide future research orientations in a particular field.

CiteSpace (http://cluster.cis.drexel.edu/~cchen/citespace) is java-based software for scientific mapping that visualizes and conceptualizes research fields as “scientific maps,” recognizes high citation, and recognizes and forecasts upcoming research trends (18). VOSviewer (www.vosviewer.com/) is novel software for building Internet-based “data maps” and visualizing and exploring them subsequently (19). VOSviewer and CiteSpace can reflect directly the development of a research field by presenting numerous data in the form of “knowledge maps,” including the productivity of authors and institutions, the geographical distribution of regions, and the results of collaborative relationships. Thus, VOSviewer and CiteSpace are used widely in applications in various fields (19, 20).

Studies providing an overview of treatment for CRS utilizing bibliometric and visualization methods to investigate the longitudinal and cross-sectional characteristics, trends, and multiple ramifications of this topic have not been published. Therefore, we endeavored to identify collaborative networks among authors, institutions and nations. We also aimed to explore key contributors to the field in the last 20 years, and identify potential hotspots and research trends from different perspectives. These analyses portrayed a “bottom–up” view with historical and prospective viewpoints. This strategy can bring new insights to scholars, helping them to draft and manage their scientific studies, and assist rhinologists in gaining a wide grasp of the macro- and micro- aspects of the entire domain of CRS knowledge.

## Materials and Methods

### Sources of Data and Strategies for Searching

**Figure 1:**
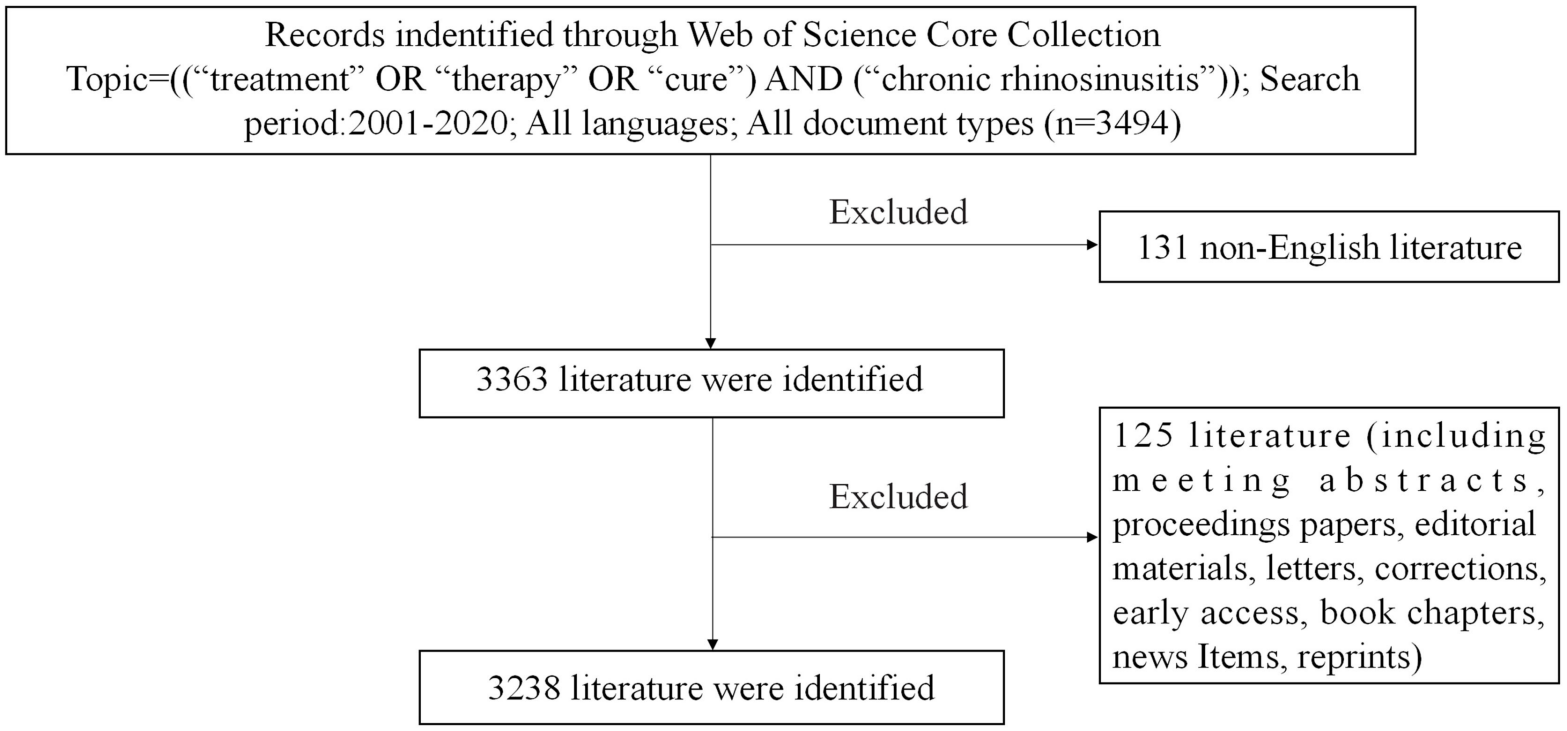
Flowchart showing our search strategy.

The bibliometric study was carried out using the Citation Index-Expanded (SCI-E) and Social Sciences Citation Index (SSCI) from the Web of Science Core Collection (WoSCC). The search terms were Topic = (“treatment” OR “therapy” OR “cure”) AND (chronic rhinosinusitis). We searched the WoSCC database extensively for relevant data between 2001 and 2020, and only original research articles and review articles were included. The only language allowed was English. Other document types and non-English articles were excluded. All data downloads and document searches were completed on 1 October 2021 to avoid a bias induced by regular updating of databases. The detailed search procedure is depicted in Figure 1. Two academics studied the data separately. Conflicts were settled by discussion or by enlisting the assistance of other specialists. We noted information on titles, abstracts, authors, institutions, countries, journals, references, and citations.

### Bibliometric Analysis

WoSCC data were converted to text before being loaded into the analysis software. VOSviewer 1.6.16 (Leiden University, Leiden, the Netherlands), CiteSpace 5.8. R3, 64-bit (Drexel University, Philadelphia, PA, USA), and a bibliometric online analysis platform (http://bibliometric.com/) were used to locate co-cited articles, keywords, countries, institutions, journals, authors, and network features of “keyword bursts,” as well as to present the results visually. The Journal Citation Report 2020 included the H-index, impact factor, and category quartiles. We queried the H-index (which is considered an important indicator to determine the scientific impact of a journal), author or country (21).

We used CiteSpace to undertake a series of analyses of publications to find research hotspots in CRS therapy. This included incorporation of the publishing institution, co-cited references, and most relevant keywords. On the network visualization map we constructed, the nodes reflected the examined items, with bigger nodes representing items that occurred more often. In addition, we analyzed the centrality score using Citespace. The centrality score evaluates the relevance of network nodes, with greater centrality representing a more significant node (22).

VOSviewer can be used to create “scientific knowledge networks” that illustrate the evolution of research fields, institutional collaboration, and foreshadow future research hotspots. We utillzed VOSviewer to assess visually the co-occurrence of terms and to construct density maps. Co-occurrence analysis in VOSviewer can be employed to classify keywords into different “clusters” represented by different colors. The clustering analysis of study hotspots can be visualized, and the keyword co-occurrence network can forecast growing trends.

## Results

### Annual Outputs and Growth Trends

**Figure 2:**
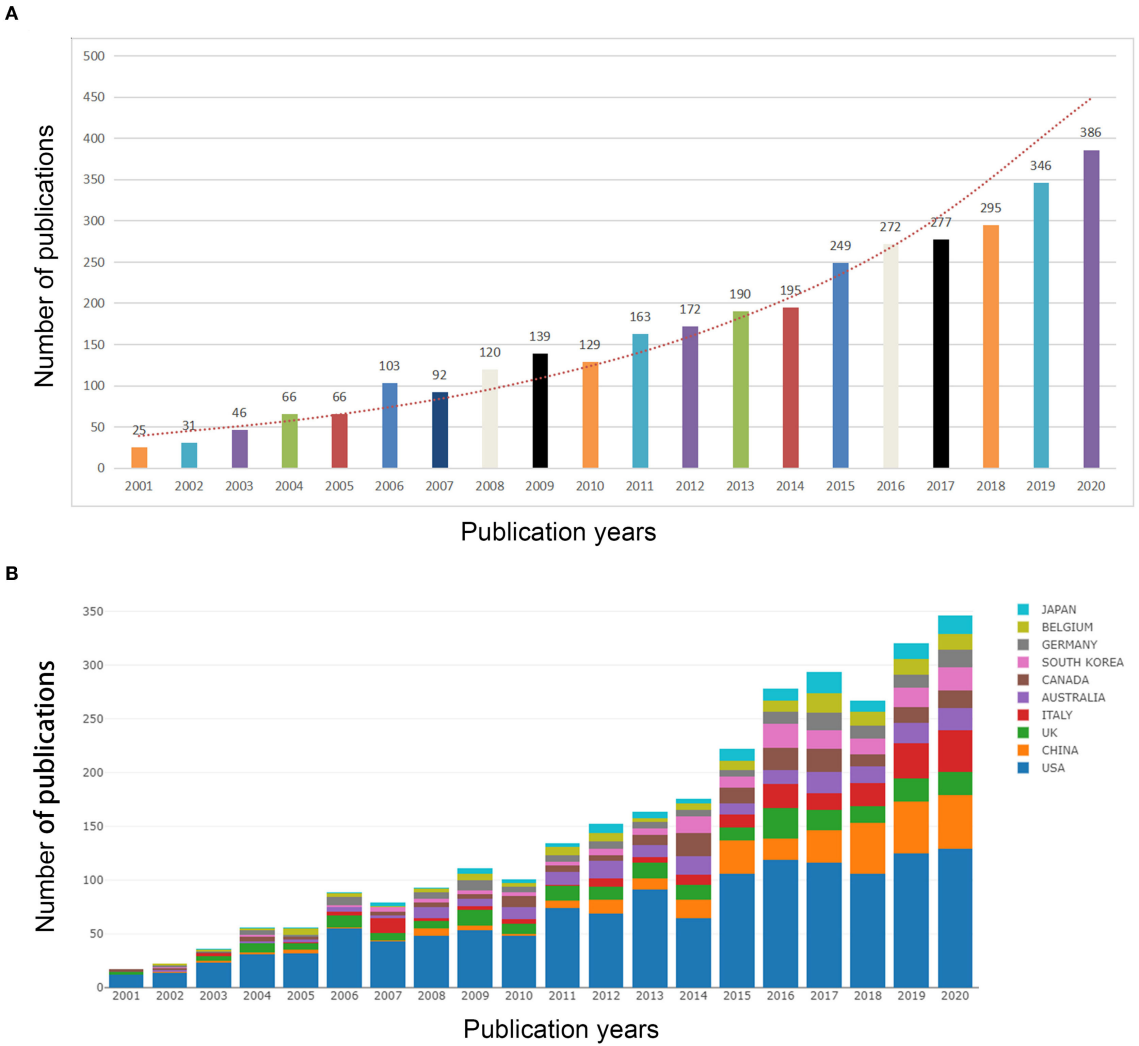
Trends in the number of publications **(A)** and the top-10 countries/regions **(B)** of CRS-treatment research from 2001 to 2020.

A total of 3238 original articles on CRS therapy were published between 2001 and 2020. Research outputs linked to CRS therapy exhibited an overall rising trend from 2001 to 2020 (Figure 2A). The number of articles published by US scholars reached an all-time high in the past two decades (Figure 2B). The number of publications on CRS treatment has increased considerably since 2015, with >15-times as many published in 2020 as published in 2001. From 2015 to 2020, CRS research activity peaked, with 2,246 papers being published in 4 years, accounting collectively for 48.9% of the overall number of papers.

### Distribution of Countries/Regions and Institutions

**Figure 3:**
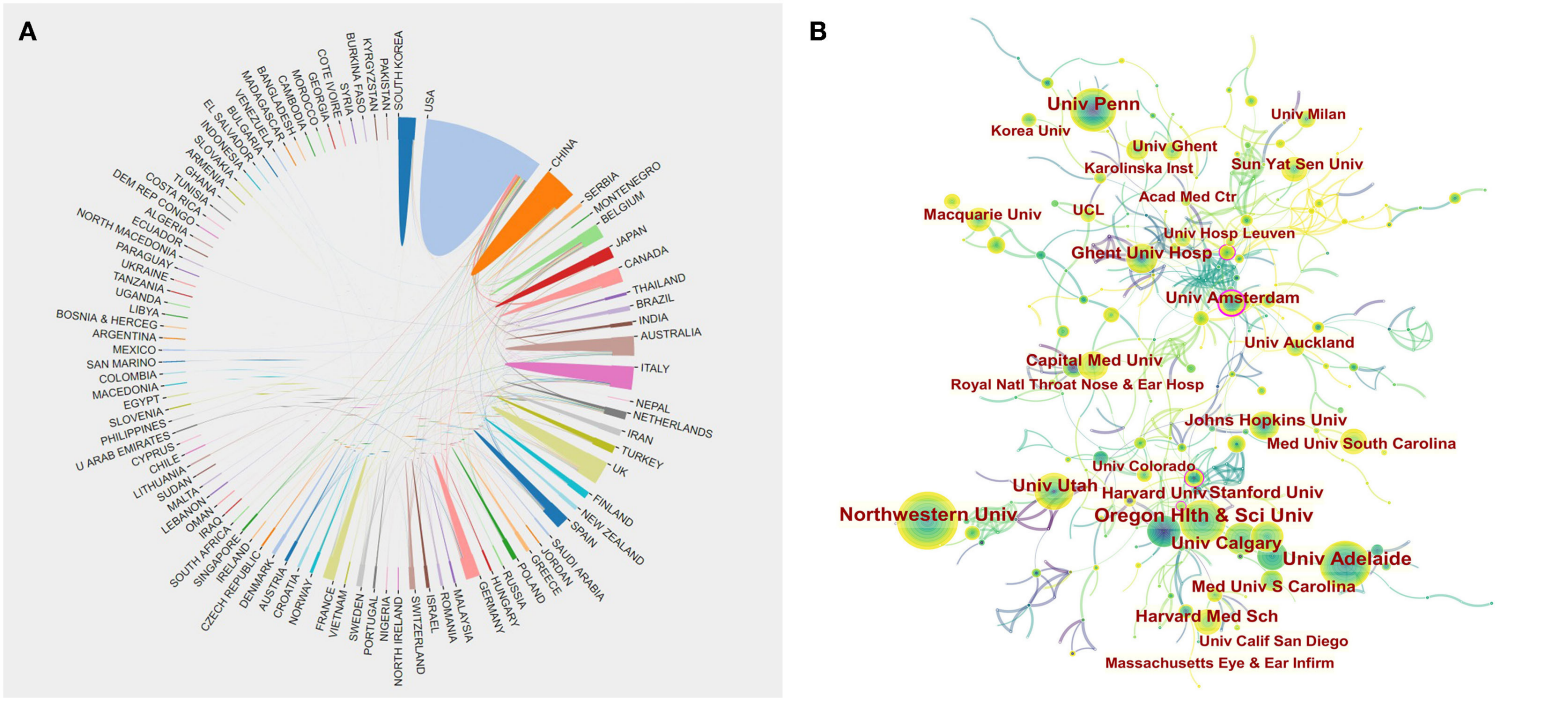
Co-operation of countries/regions **(A)** and institutions **(B)** contributing to publications on CRS-treatment research from 2001 to 2020.

Figure 3A displays the country-collaboration network of research into CRS treatment. The top-10 contributing countries are shown in Table 1. Most publications came from the USA (1390), followed by China (242), the UK (230), Italy (202), and Australia (199). Among the top-10 countries, the USA was the most prolific producer of CRS research, publishing >40% of studies. The centrality score is a metric that assesses the significance of network nodes. Centrality analysis showed that the USA (0.61) was at the network core, followed by Canada (0.09), and Italy (0.03). Higher centrality in a collaborative network correlated to more frequent cooperation. The low density of the country-based research-network map indicated largely independent research teams, which underlined the need for further collaboration.

**Table 1:** Ranking of top-10 countries that have published the most article on CRS-treatment research from 2001 to 2020.

**Rank**	**Article counts**	**Centrality score**	**Country**
1	1,390	0.61	USA
2	242	0.00	China
3	230	0.00	UK
4	202	0.03	Italy
5	199	0.03	Australia
6	182	0.09	Canada
7	159	0.00	South Korea
8	139	0.00	Germany
9	136	0.00	Belgium
10	126	0.00	Japan

The institution-collaboration network (Figure 3B) revealed the top-10 institutions, including the USA institutions Harvard University (124), University of California system (124), Northwestern University (102), Oregon Health and Science University (101), and University of Pennsylvania (101) (Table 2). The universities with the highest centrality score were Harvard University (0.08), University of California system (0.05), and University of Adelaide (0.62).

**Table 2:** Ranking of top-10 institutions that have collaborated the most on CRS-treatment research from 2001 to 2020.

**Rank**	**Article count**	**Centrality score**	**Institution**	**Country**
1	124	0.08	Harvard University	USA
2	124	0.05	University of California system	USA
3	102	0.00	Northwestern University	USA
4	101	0.03	Oregon Health and Science University	USA
5	101	0.02	University of Pennsylvania	USA
6	100	0.05	Ghent University	Belgium
7	92	0.00	Medical University of South Carolina	USA
8	88	0.05	University of Adelaide	Australia
9	88	0.01	University of London	UK
10	87	0.02	Feinberg School of Medicine	USA

### Contributions of Authors

**Figure 4:**
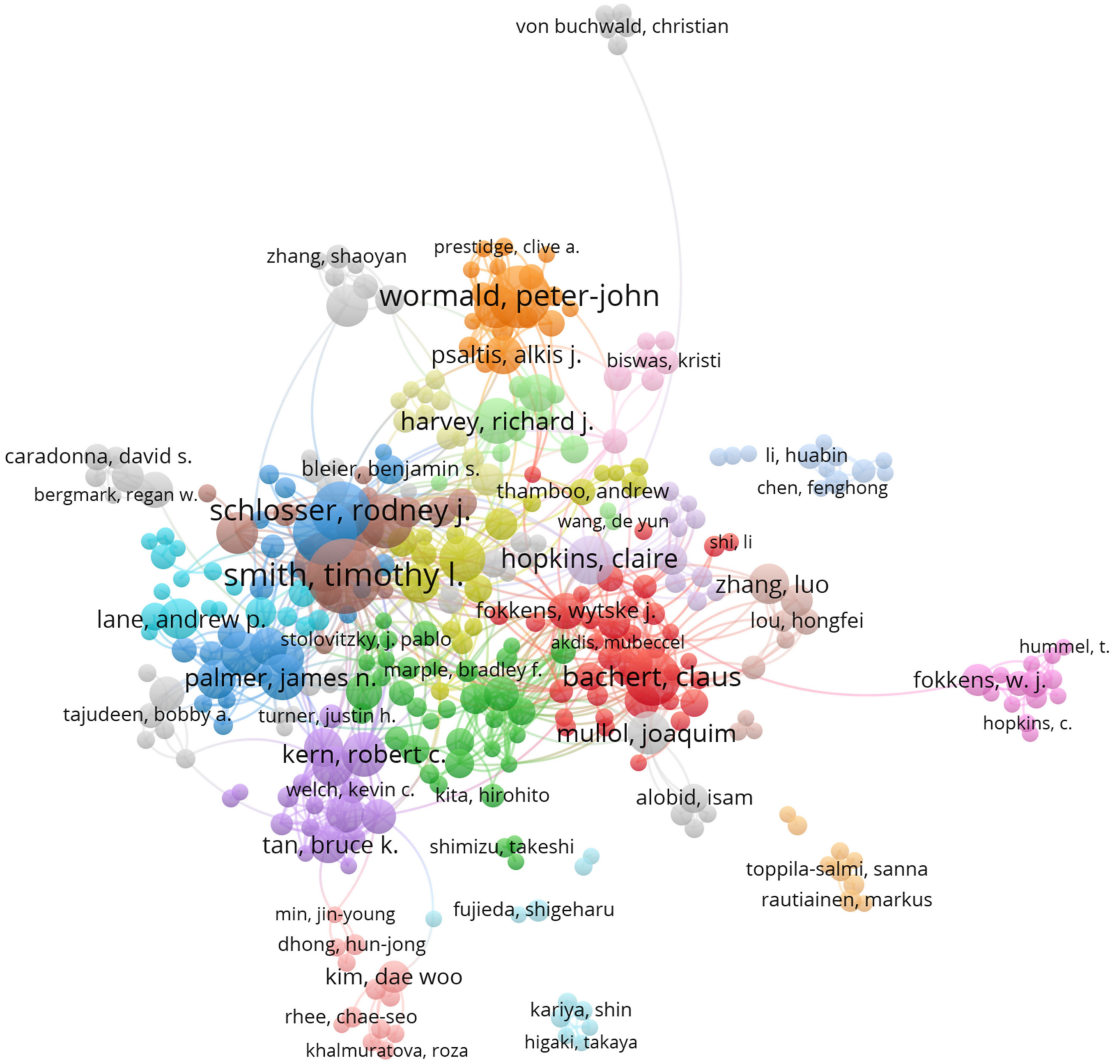
Joint mapping of productive authors in CRS-treatment research from 2001 to 2020.

A visualization map of co-authorship can be used to identify research organizations with the greatest influence and potential collaborators, as well as to help researchers form collaborative ties. Authors with ≥5 publications and ≥500 citations were visualized using VOSviewer (Figure 4). Due to overlapping names, certain names may not be seen. Closed circles indicate “active” authors with close research partnerships.

We discovered that 10,623 authors published articles on the topic of CRS from 2001 to 2020. Table 3 lists the top-10 most productive writers over the research period. T. L. Smith of Oregon Health and Science University (98 publications; 3,700 citations) had the most manuscripts published, followed by C. Bachert of Ghent University Hospital (81 publications; 6,469 citations). R. J. Schlosser of the Medical University of South Carolina ranked first in terms of the centrality score (0.10).

**Table 3:** Ranking of top-10 most-published authors on CRS-treatment research from 2001 to 2020.

**Rank**	**Author**	**Article count**	**Centrality score**	**Total number of citations**	**Average number of citations**	**H-index**
1	Smith TL	98	0.01	3,700	37.76	35
2	Bachert C	81	0.04	6,469	79.86	43
3	Wormald PJ	79	0.03	3,084	39.04	31
4	Soler ZM	68	0.01	2,124	31.24	28
5	Schlosser RJ	62	0.10	2,291	36.95	26
6	Mace JC	57	0.03	1,832	32.14	22
7	Rudmik L	54	0.02	2,499	46.28	28
8	Mullol J	52	0.03	3,845	73.94	28
9	Fokkens WJ	51	0.06	3,535	69.31	33
10	Hopkins C	51	0.01	2,742	53.76	25

### Journal Analyses

The features of the top-10 most active journals are shown in Table 4. Most of the publishers of these periodicals were located in the USA. The highest number of CRS-related articles was published by *International Forum of Allergy & Rhinology, American Journal of Rhinology & Allergy*, and *Laryngoscope*. Several high-impact-factor articles on CRS therapy were published in *Journal of Allergy and Clinical Immunology*. In addition, the highest average number of citations (94.76) and H-index (44) were achieved by *Journal of Allergy and Clinical Immunology*. The Journal Citation Report quartile Q1 included *International Forum of Allergy & Rhinology, Rhinology*, and *Journal of Allergy and Clinical Immunology*. Q2 contained *American Journal of Rhinology & Allergy, European Archives of Oto-Rhino-Laryngology, Current Allergy and Asthma Reports, Otolaryngology-Head and Neck Surgery*, and *American Journal of Rhinology*. *Laryngoscope* and *Current Opinion in Otolaryngology & Head and Neck Surgery* were ranked as Q3.

**Table 4:** Ranking of top-10 journals for the number of articles published on CRS-treatment research from 2001 to 2020.

**Rank**	**Journal**	**Article count**	**Country**	**Journal citation reports (2020)**	**Impact factor (2020)**	**Total number of citations**	**Mean number of citations**	**H-index**
1	International Forum of Allergy & Rhinology	351	USA	Q1	3.858	7,299	20.79	40
2	American Journal of Rhinology & Allergy	270	USA	Q2	2.467	4,649	17.22	36
3	Laryngoscope	179	USA	Q3	3.325	6,089	30.91	42
4	Rhinology	144	Netherlands	Q1	3.681	4,062	28.21	33
5	European Archives of Oto-Rhino-Laryngology	110	Germany	Q2	2.503	1,397	12.70	21
6	Current Allergy And Asthma Reports	99	USA	Q2	4.806	1,657	16.74	23
7	Otolaryngology-Head And Neck Surgery	89	USA	Q2	3.497	3,636	40.85	30
8	Journal of Allergy And Clinical Immunology	74	USA	Q1	10.793	7,012	94.76	44
9	Current Opinion in Otolaryngology & Head and Neck Surgery	69	USA	Q3	2.064	1,035	15.00	20
10	American Journal of Rhinology	68	USA	Q2	3.467	2,782	40.91	32

### Cluster Analysis of Keyword Co-occurrence Related to Research Hotspots

**Figure 5:**
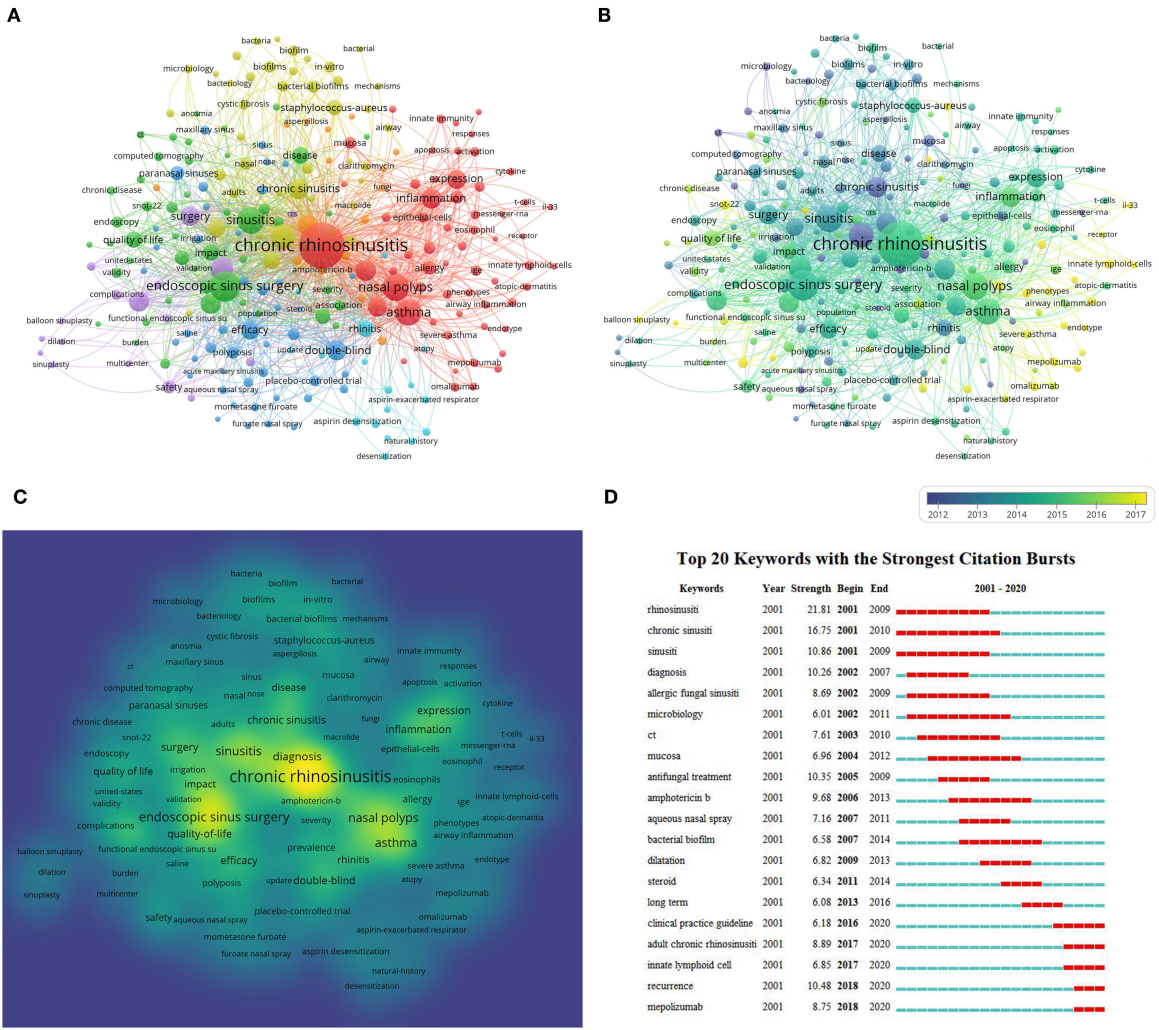
Co-occurrence analysis of global research on CRS treatment based on the WoSCC database from 2001 to 2020. **(A)** Mapping of keywords in the research field. **(B)** Distribution of keywords according to the chronological order of appearance. **(C)** Distribution of keywords according to the mean frequency of appearance. **(D)** Keywords with the strongest citation bursts in CRS-treatment research.

VOSviewer was used to search the titles and abstracts of the 3,238 retrieved articles for keywords. The result was a map of 243 terms (a total of 8,373), each with ≥20 occurrences, which were grouped as five clusters (Figure 5A). In the map, the high-frequency keywords were “CRS” (1791), “ESS” (718), “sinusitis” (561), “nasal polyps” (541), “asthma” (525), “rhinosinusitis” (446), “management” (376), “diagnosis” (304), “outcomes” (295), and “quality-of-life” (289). The terms with similar research subjects were combined under the same catalog, with five main clusters: clinical features, pathogenesis, diagnosis, treatment, and pathophysiology of CRS.

The keyword distribution in terms of order of occurrence was seen using VOSviewer (Figure 5B). The number of times a keyword occurred was determined by the color of the area. Before 2010, most studies concentrated on the themes of “clinical characteristics” and “clinical treatment,” whereas the latest trends identified indicated that “precision medicine” and “phenotypes” might become future research emphases.

Meanwhile, VOSviewer was utilized to measure the frequency of keywords to calculate their density, which was presented as a density map (Figure 5C). The “warmer” the hue (toward yellow), the higher was the density. In a particular field, research hotspots tend to form in locations with higher grayscale values.

### Detection of Keyword Bursts

Keyword bursts between 2001 and 2020 were detected on the basis of examination of 3,238 articles included in the WoSCC database (Figure 5D). The chronology is displayed by a blue line that cuts through a year. The burst period is shown by a red reflection line that marks the start year and finish year, as well as the timespan of the citation burst. We eliminated terms that had little or no research value so that we could focus on representing the research trends of CRS treatment. From 2001 to 2010, “rhinosinusitis” had the highest burst strength (21.81). Between 2011 and 2020, “recurrence” had the highest burst strength (21.81), followed by “adult CRS” (8.89) and “mepolizumab” (8.75).

### Analyses of Co-cited References

**Figure 6:**
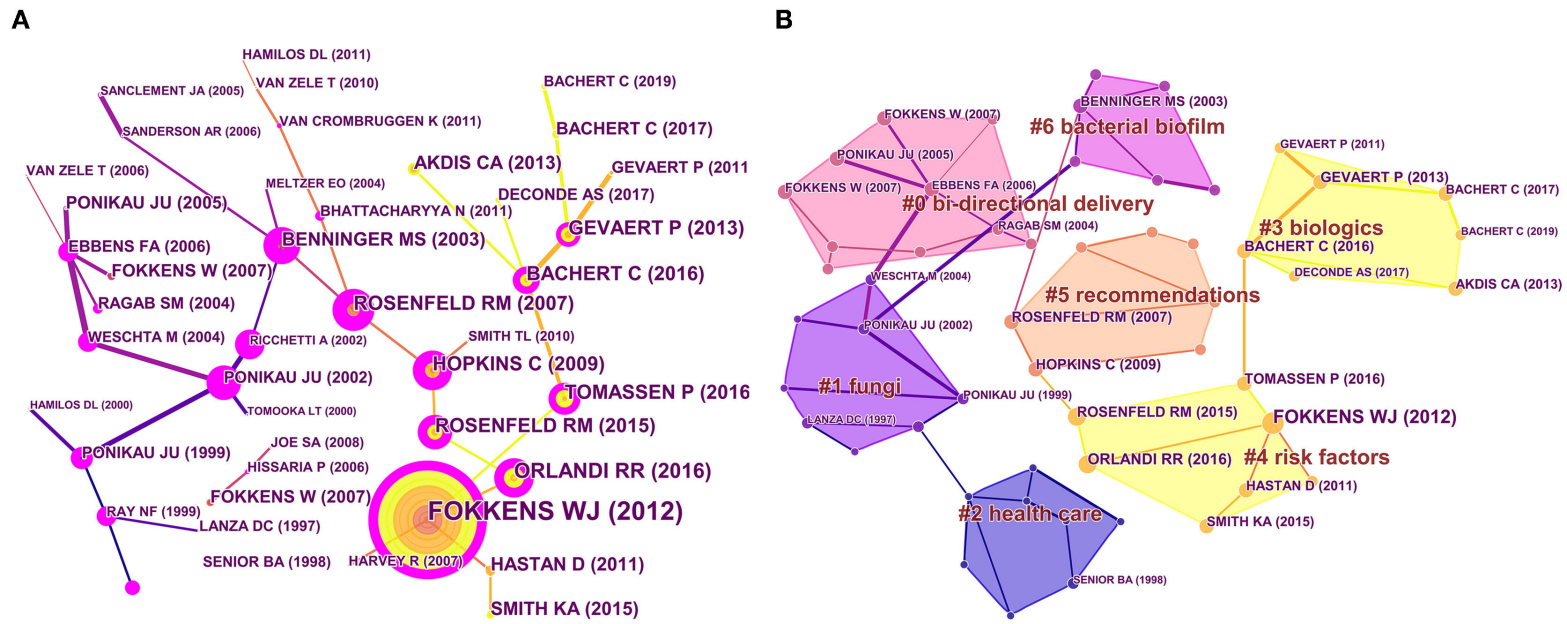
Co-cited references map **(A)** and clustered network map of co-cited references **(B)** on CRS-treatment research from 2001 to 2020.

From 3,238 articles, 59,440 cited references were submitted for analyses of co-citation correlation, and a cluster network map was created from the results. Figure 6A presents the visualized network of the co-cited articles, which has 53 nodes and 54 links. A referenced article is represented by each node. The links between nodes show the frequency with which the same article is quoted. The node diameter is proportional to the total number of co-citations of the article. The nodes (which have a thick purple ring around them) can be used to connect the stages of the growth of a field. An “explosion” of citations is shown by a red ring. Then, by creating a hierarchical order of the co-cited articles created in the co-citation network, research hotspots can be found.

**Figure 7:**
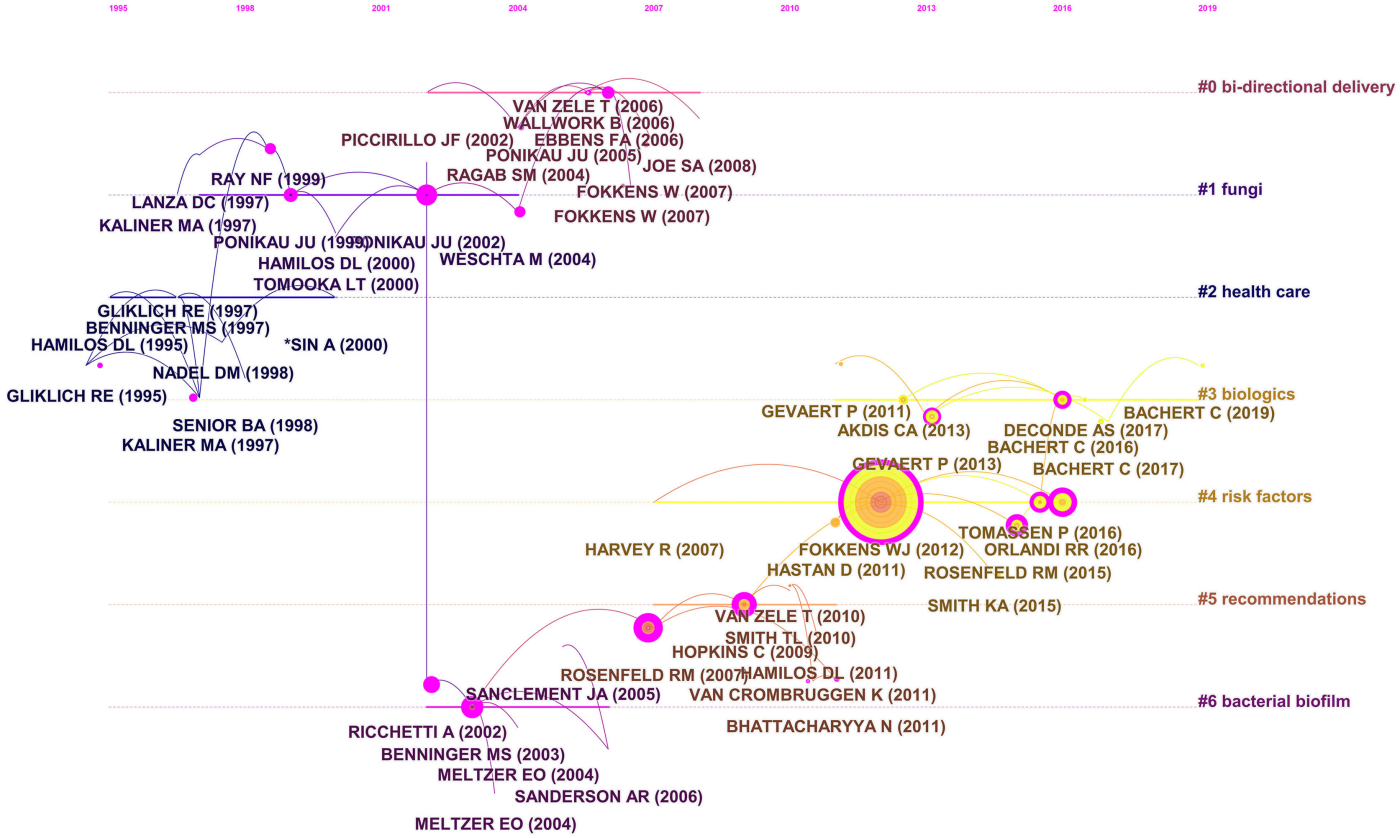
Timeline view of co-cited clusters with cluster labels.

**Figure 8:**
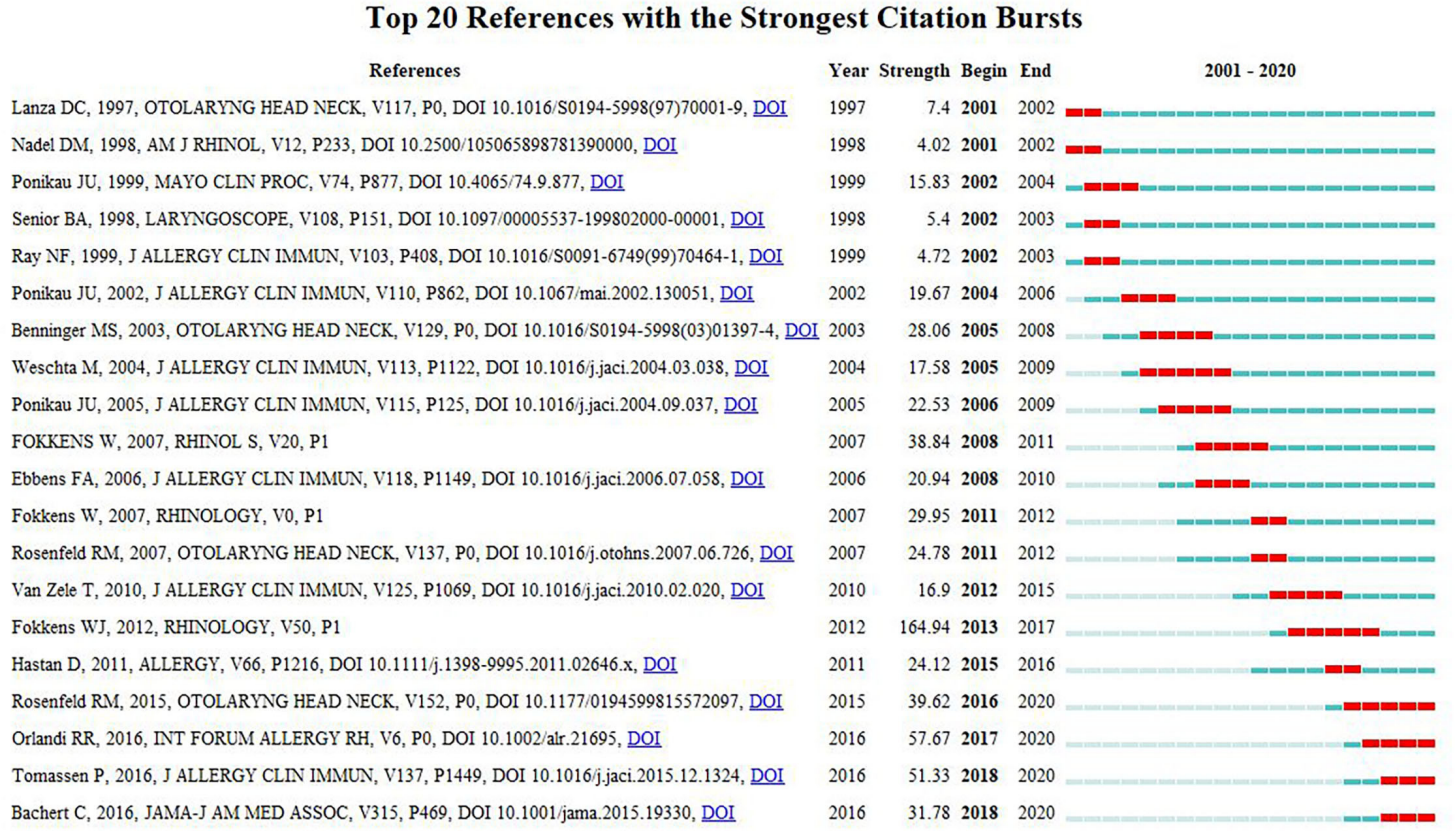
Top 20 references with the strongest citation bursts.

“Bi-directional delivery,” “fungi,” “health care,” “biologics risk factors,” “recommendations,” and “bacterial biofilm” were among the 11 key clusters of co-cited references (Figure 6B). The timeline view of the clustering plot is shown in Figure 7, which aids identification of emerging research hotspots in CRS treatment. The top-10 co-cited articles are listed in Table 5. The study by Hall-Stoodley et al. (23) had the most citations in *Cellular Microbiology* (653 citations), followed by Fokkens et al. (24) in *Rhinology* (558 citations), and Rosenfeld et al. (25) in *Otolaryngology-Head and Neck Surgery* (512 citations). Figure 8 shows the top-20 references with the strongest citation bursts. Most of the references with citation bursts were from publications on otorhinolaryngology or allergology, indicating that allergology is a popular topic in the treatment of CRS.

**Table 5:** Top-10 most co-cited references on CRS-treatment research from 2001 to 2020.

**Rank**	**Title**	**Author**	**Year**	**Journal**	**Citation frequency**
1	Evolving concepts in biofilm infections	Hall-Stoodley L	2009	Cellular Microbiology	653
2	European position paper on rhinosinusitis and nasal polyps 2012	Fokkens WJ	2012	Rhinology	558
3	Clinical Practice Guideline (Update): Adult Sinusitis	Rosenfeld RM	2015	Otolaryngology-Head And Neck Surgery	512
4	International Consensus Statement on Allergy and Rhinology: Rhinosinusitis	Orlandi RR	2016	International Forum of Allergy & Rhinology	468
5	Cystic fibrosis genetics: from molecular understanding to clinical application	Cutting GR	2015	Nature Reviews Genetics	433
6	Allergic bronchopulmonary aspergillosis: review of literature and proposal of new diagnostic and classification criteria	Agarwal R	2013	Clinical And Experimental Allergy	430
7	Effect of Subcutaneous Dupilumab on Nasal Polyp Burden in Patients With Chronic Sinusitis and Nasal Polyposis A Randomized Clinical Trial	Bachert C	2016	Jama-Journal of The American Medical Association	410
8	Endotypes and phenotypes of chronic rhinosinusitis: A PRACTALL document of the European Academy of Allergy and Clinical Immunology and the American Academy of Allergy, Asthma & Immunology	Akdis CA	2013	Journal of Allergy And Clinical Immunology	366
9	Aspirin-induced asthma: Advances in pathogenesis, diagnosis, and management	Szczeklik A	2003	Journal of Allergy And Clinical Immunology	366
10	Mepolizumab, a humanized anti-IL-5 mAb, as a treatment option for severe nasal polyposis	Gevaert P	2011	Journal of Allergy And Clinical Immunology	322

**Figure 9:**
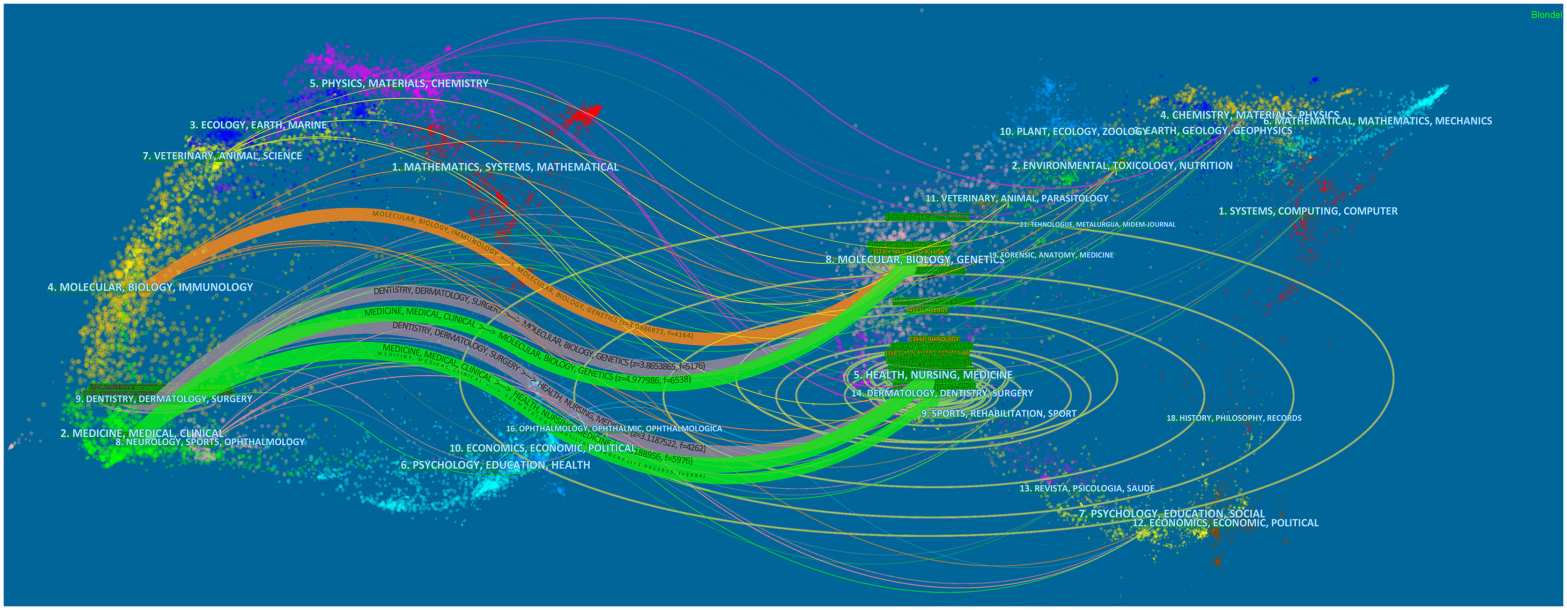
The dual-map overlay of journals on CRS-treatment.

The distribution of links between journals is displayed in a dual-map overlay of journals, with citing journals on the left and cited journals on the right. The stated relationships are indicated by the colored routes connecting them. Five main citation paths are present in Figure 9: two gray paths, two green paths, and one orange path. Studies published in Molecular/Biology/Genetic and Health/Nursing/Medicine journals are, in general, mentioned by Dentistry/Dermatology/Surgery. The green path means that the studies published in Molecular/Biology/Genetic and Health/Nursing/Medicine journals are, in general, cited by Medicine/Medical/Clinical journals, as indicated by the green route. Studies published in Molecular/Biology/Genetics journals are cited for studies in Molecular/Biology/Immunology journals, as shown by the orange route.

## Discussion

CRS is a widespread and challenging clinical issue. It has diverse phenotypes with different potential mechanisms that contribute to the persistence or recurrence of nasal polyps. The primary inflammatory patterns of CRS are believed to impact the clinical features and responses to pharmacological and surgical interventions (26). Despite the efficacy of available therapies, poor control of symptoms, frequent recurrence of nasal polyps postoperatively, and the side-effects associated with long-term corticosteroid use are critical concerns that should be addressed (27). Therefore, a general overview of current developments in global research of CRS treatment is required.

This was the first application of quantitative and qualitative bibliometric methods to CRS management. It involved 3,238 research papers retrieved from databases. We analyzed the bibliometric output of articles in the field of CRS therapy worldwide. We revealed the major research hotpots and trends between 2001 and 2020. Based on the growth curve, we speculate that increasing numbers of researchers are showing interest in CRS treatment. The latter remains a research hotspot, with the number of publications related to CRS treatment expected to keep increasing.

The USA leads the way by contributing >40% of papers in CRS treatment. Most of the top-10 institutions originate from the USA, which has stimulated advancement of research on CRS treatment. On the one hand, this trend reflects the mature environment of medical research and health in the USA but, on the other hand, it reflects an urgent demand for efficacious CRS treatments. Moreover, the USA had the highest centrality score and most active cooperation with other countries. Many publications were provided by South Korea, the UK, and Germany, but they might have fewer collaborations with other countries, as evidenced by their lower centrality score. We suggest that countries with a lower centrality score should strengthen international exchange and cooperation with other countries to establish a good partnership, particularly with the pioneering countries in CRS research, which will accelerate their progress in this field. Harvard University, University of California system, and Ghent University have the most collaborations with other institutions, which is a worthwhile lesson for those institutions that rarely interact with each other.

T. L. Smith had the most publications in the field of CRS therapy. C. Bachert, P. J. Wormald, Z. M. Soler, and R. J. Schlosser were the most productive authors in the past two decades. A distinct geographic pattern of global investigators in CRS treatment became evident. Most scholars were working in Europe and the USA, and these authors were working mainly in the otolaryngology departments of their university-affiliated hospital.

The journals with the maximum number of publications on CRS treatment were *International Forum of Allergy & Rhinology, American Journal of Rhinology & Allergy, Laryngoscope, Rhinology*, and *European Archives of Oto-Rhino-Laryngology*. These publications are world leaders in the field of otolaryngology and allergy. This trend suggests that journals specializing in rhinology and allergy are more attractive to scholars researching the treatment of CRS. It also reflects that CRS-treatment is a hot topic of research in rhinology and allergology. The top-10 co-cited references for the period 2001–2020 demonstrated that scholars were paying more attention to the clinical administration of CRS. Notably, the “2012 European Position Paper on Rhinitis and Nasal Polyps” published by Fokkens et al. was recognized as a treatment guideline by rhinologists worldwide. A dual-map overlay provides a macroscopic view of the evolution of research content at the discipline level In Figure 8, the dual-map overlay of journals indicates the disciplinary distribution of academic journals. Immunology, molecular, biology, and genetics are the fundamental and core subjects of CRS treatment. In consideration of the five major pathways in the map, research into CRS treatment has begun to translate major basic research into clinical studies.

The top-10 high-frequency keywords in co-occurrence cluster analyses demonstrated that the potential pathophysiological mechanism, optimal treatments, and outcome evaluation of multi-treatment regimens continue to be top topics. Burst keywords indicate emerging trends and research frontiers. Five frontiers of CRS research were identified: “clinical practice guideline” (2016–2020), “adult CRS” (2017–2020), “innate lymphoid cell” (2017–2020), “recurrence” (2018–2020), and “mepolizumab” (2018–2020).

During recent years, CRS recurrence has become a hotspot in the field of CRS treatment. CRS is a highly heterogeneous disease with complex pathophysiologic mechanisms. CRSwNP has been a focus of research in the field of rhinology due to its relatively severe clinical symptoms, poor therapeutic control, and high prevalence of postoperative recurrence. Although cure or control has been achieved in some CRSwNP patients, a proportion of patients require reoperation, and longer follow-up is accompanied by the higher risks of recurrence and reoperation. A meta-analysis of the efficacy of endoscopic procedures showed a reoperation prevalence of 19% in 34,220 patients in 45 studies. The reoperation prevalence increased to 23–29% if patients had comorbid asthma, a history of previous surgery, allergic fungal sinusitis, or aspirin-aggravated respiratory disease (28). One study in the USA reported a mean follow-up of 4.4 years in 338 patients and a reoperation prevalence of 25% (29). A recent multicenter study in Japan showed a mean follow-up of 3.3 years and a recurrence prevalence of 23% in patients with CRSwNP (30). In recent years, precision medicine has been recognized increasingly as a direction to optimize the care of CRS patients. Different phenotypes of CRS have been described based on the severity and duration of symptoms, atopy status, degree of control, comorbidities, and accompanying nasal polyps in CRS. Endotype-driven therapy is a key component of precision medicine, especially in patients with advanced uncontrolled disease (31). Monoclonal antibodies could be new therapy if patients with the phenotype and endotypes that will benefit most from these treatments can be identified (8).

In recent years, scholars have recognized the need for reliable biomarkers to provide predictive information about the diagnosis, endotype, treatment response, and risk of future recurrence of CRS. Driven by advances in basic research, various biomarkers of CRS have been explored. In terms of pathogenesis, CRSwNP biomarkers appear to fall into six primary categories: eosinophils, type-2 cytokines, immunoglobulins, remodeling factors, nasal nitric oxide (nNO), along with molecules involved in corticosteroid responsiveness and olfactory loss (32). Biomarkers of CRSwNP can be derived from various sources: nasal secretions, nasal biopsies, exhaled breath, and peripheral blood. Eosinophil counts in the nasal mucosa have proven particularly valuable for endotyping, assessing disease severity, as well as predicting responsiveness to corticosteroids and surgical outcomes (33). Blood eosinophilia may serve as a substitute for eosinophilic inflammation in tissue, but its utility is limited. Type-2 cytokines have been identified as potential therapeutic targets. Moreover, expression of matrix metalloproteinase-9 has been correlated with the quality of healing after sinus surgery (34). As a biomarker of eosinophilic inflammation in the upper airway, nNO appears to fill a gap in the non-invasive measurement of sinus ostial patency (35). Furthermore, recent data suggest involvement of some promising biomarkers in corticosteroid resistance and olfactory dysfunction (36). However, rigorous validation utilizing large cohort studies is necessary before these biomarkers can become mainstream in clinical practice.

Judging from the timeline of the cluster map, biologic agents have become an emerging focus in CRS treatment during recent years. Current treatments are struggling to control disease progression in intractable CRSwNP, so exploring novel therapeutic modalities is an urgent need. Recently, a series of biologic agents, monoclonal antibodies, have entered the field of CRSwNP treatment. They include mepolizumab, omalizumab, and dupilumab, and mainly target the effector products of type-2 inflammation (37). Academic organizations worldwide have published guidelines or expert consensuses on the treatment of CRSwNP with biologic agents (38–40). Clinical studies have shown that biologic agents can relieve nasal congestion by shrinking nasal polyps while enhancing olfaction and improving quality of life. It has been have reported that all three biologics, dupilumab, omalizumab, and mepolizumab, can reduce the size of nasal polyps and improved nasal symptoms, with dupilumab having the best efficacy and omalizumab and mepolizumab being equivalent, and all three having similar effects on improving quality of life (41, 42). These effects decrease use of oral glucocorticoids and need for reoperation, thereby providing new treatment for refractory CRSwNP (39, 40). However, application of biologic agents in the clinical setting for CRS treatment has three main limitations. First, biologic agents are not efficacious in all patients with CRS. Some studies have reported that the percentage of patients with CRS who respond to biologic therapy is 50–70% (41). Second, biologic agents mainly target the effector products of type-2 inflammation, and cannot be applied to CRSsNP (43). Furthermore, the high cost hampers the widespread application of biologics. Notably, biologics also have some potential side effects, and they require continuous use and a longer treatment course. Most commonly reported adverse events (AEs) for dupilumab were cough, bronchitis, arthralgia, and injection-site reactions, nasopharyngitis, headache, worsening of asthma, epistaxis, and injection-site erythema (44). AEs for mepolizumab with >5% incidence were oropharyngeal pain, back pain, influenza, and pyrexia (45). Randomized phase III trials showed that most common AEs for omalizumab were headache, injection-site reactions, arthralgia, dizziness, and upper abdominal pain (46).

### Limitations

This is the first time that bibliometric analysis has been used for research into CRS treatment, but four limitations should be considered. First, continuous updating of the changes in the database might have led to variations between search results and the number of actual publications included. Second, considering that the WoSCC database is one of the most extensive and well-recognized global resources (17, 47), the information on these publications was only from WoSCC, so some publications from other databases may have been missed. Third, the omission of books/chapters/letters and the consideration of articles published in English alone may have led to some biases in our analyses. Fourth, when comparing countries of different sizes and populations, a per capita analysis was not considered, and this omission could have produced a research bias. Lastly, the wealth of different countries is a potential cause of research bias, as it would limit investment in health research. Nevertheless, we believe that this bibliometric analysis presents the overall situation and general trend in CRS treatment. This visualization-based analysis of publications has laid the foundation for researchers to understand rapidly the hotspots and trends in CRS-treatment research.

## Conclusion

This bibliometric study analyzed publications on CRS treatment worldwide. Throughout the past two decades, the number of publications in the field of CRS treatment has increased year-by-year. This field has also stimulated a great deal of scholarly interest, and will remain a hotspot in the future. Articles published in journals specializing in rhinology and allergy received more attention than generalist journals. The USA contributed >40% of CRS publications worldwide, which has contributed significantly to the development of the field. Therefore, collaboration between countries, institutions, and research teams should be strengthened. CRS recurrence and use of biologic agents are the research hotspots in the field of CRS treatment.

## Data Availability Statement

The raw data supporting the conclusions of this article will be made available by the authors, without undue reservation.

## Author Contributions

FZ and GY conceived the study. FZ, TZ, and YJ collected the data. FZ and YM re-examined the data. FZ, ZX, and MZ analyzed the data. FZ wrote the manuscript. GY and TZ reviewed and revised the manuscript. All authors contributed to the article and approved the submitted version.

## Funding

This work was supported by the Science and Technology Foundation of Guizhou Province (D2011160), National Natural Science Foundation Cultivation Project of Affiliated Hospital of Guizhou Medical University (gyfynsfc [2020]-7), and Science and Technology Fund Project of Guizhou Provincial Health Commission (gzwkj2022-155).

## Conflict of Interest

The authors declare that the research was conducted in the absence of any commercial or financial relationships that could be construed as a potential conflict of interest.

## Publisher's Note

All claims expressed in this article are solely those of the authors and do not necessarily represent those of their affiliated organizations, or those of the publisher, the editors and the reviewers. Any product that may be evaluated in this article, or claim that may be made by its manufacturer, is not guaranteed or endorsed by the publisher.
